# AKTivation of PI3K/AKT/mTOR signaling pathway by KSHV

**DOI:** 10.3389/fimmu.2012.00401

**Published:** 2013-01-07

**Authors:** Aadra P. Bhatt, Blossom Damania

**Affiliations:** ^1^Lineberger Comprehensive Cancer Center, University of North Carolina at Chapel HillChapel Hill, NC, USA; ^2^Department of Microbiology and Immunology, University of North Carolina at Chapel HillChapel Hill, NC, USA

**Keywords:** Akt, KSHV, mTOR, PI3K, B cells

## Abstract

As an obligate intracellular parasite, Kaposi sarcoma-associated herpesvirus (KSHV) relies on the host cell machinery to meet its needs for survival, viral replication, production, and dissemination of progeny virions. KSHV is a gammaherpesvirus that is associated with three different malignancies: Kaposi sarcoma (KS), and two B cell lymphoproliferative disorders, primary effusion lymphoma (PEL) and multicentric Castleman’s disease. KSHV viral proteins modulate the cellular phosphatidylinositol-3-kinase (PI3K)/AKT/mammalian target of rapamycin (mTOR) signaling pathway, which is a ubiquitous pathway that also controls B lymphocyte proliferation and development. We review the mechanisms by which KSHV manipulates the PI3K/AKT/mTOR pathway, with a specific focus on B cells.

## INTRODUCTION

Kaposi sarcoma-associated herpesvirus (KSHV; also known as human herpesvirus 8) is a human gammaherpesvirus that was discovered in Kaposi’s sarcoma (KS) biopsies in 1994 (Chang et al., 1994). Following this seminal discovery, KSHV has been found in all forms of KS, including KS associated with AIDS patients, as well as HIV-negative and transplant-associated KS. In addition to KS, which is a vascular endotheliosarcoma, KSHV is also tightly associated with two lymphoproliferative disorders: primary effusion lymphoma (PEL; Cesarman et al., 1995 and the plasmablastic variant of multicentric Castleman’s disease (MCD; Gessain et al., 1996, both arising from infection of B cells. Owing to the association with these three cancers, KSHV has been extensively studied, and the results of these studies have revealed fascinating mechanisms by which this oncogenic herpesvirus alters the infected cell in order to promote transformation and tumorigenesis.

## B LYMPHOCYTE DEVELOPMENT

B and T cells descend from a common lymphoid progenitor cell, itself derived from a hematopoietic stem cell precursor. In humans, B cell development occurs in the bone marrow, where the earliest progenitor (or pre-pro) B cell expresses germline heavy- and light-chain immunoglobulin genes (Murphy et al., 2008). As the B cell matures, movement along the bone marrow and interaction with stromal cells leads to maturation. D-J gene rearrangement occurs in early pro-B cells, and continues to V-DJ rearrangement in the late pro-B cell. These gene rearrangements create a unique variable domain in the immunoglobulin. Allelic exclusion is enforced by the pre-B cell receptor, whereby only one allele encoding the rearranged heavy chain is expressed, thereby ensuring that each B cell has specificity for a single antigen (Murphy et al., 2008). Many rounds of cell division occur during the transition of pro-B cells to the pre-B cell stage, leading to the formation of numerous small pre-B cells with a specific rearranged μ heavy-chain gene. Pre-B cells undergo light-chain gene rearrangement, which is also accompanied by allelic exclusion. Since these pre-B cells now produce both heavy- and light-chain proteins, they are classified as immature B cells, and bear intact IgM molecules on their cell surface (Murphy et al., 2008). For a review describing normal B cell development, please see Montecino-Rodriguez and Dorshkind (2012)

In addition to allelic exclusion, isotype exclusion also occurs in immature B cells, wherein the immature B cell expresses only one light chain (either λ or κ; Murphy et al., 2008. In humans, because the κ gene rearranges prior to the λ gene, many more mature B cells express the κ light chain rather than λ. The average distribution of κ to λ-bearing B cells in humans is approximately 65:35%, and aberration from this ratio is indicative of lymphoproliferative disorders, reflecting dominance of one clone (Murphy et al., 2008).

## PATHOPHYSIOLOGY OF KSHV-ASSOCIATED B CELL MALIGNANCIES

### PRIMARY EFFUSION LYMPHOMA

Primary effusion lymphoma mainly afflicts HIV-infected patients, and occurs in body cavities such as the peritoneal, pleural, and pericardial cavities (Green et al., 1995; Nador et al., 1996. Some KSHV-positive lymphomas can also present as extranodal solid masses, which may subsequently develop into an effusion. Cells have an immunoblastic appearance with a high mitotic index. KSHV-positive solid lymphomas represent an extracavitary variant of PEL (Arvanitakis et al., 1996). In PEL, every tumor cell expresses between 50 and 150 copies of the KSHV genome. The genome is found as an episome tethered to the host cell chromosome by the virus-encoded latency-associated nuclear antigen (LANA) protein (Ballestas et al., 1999; Cotter and Robertson, 1999; Schwam et al., 2000; Garber et al., 2001). Some PEL are co-infected with Epstein–Barr virus (EBV), another lymphotropic gammaherpesvirus (Cesarman et al., 1996; Nador et al., 1996).

Patients with PEL present with lymphomatous effusions within body cavities, in the absence of a solid tumor mass (Nador et al., 1996; Ambinder and Cesarman, 2007). Cells contained within the effusions are large, with abundant cytoplasm, and display morphological aspects common to both large-cell immunoblastic and anaplastic large cell lymphoma (Nador et al., 1996). Analysis of *Ig* rearrangements suggests that PEL arises from clonal expansion of an infected B cell (Green et al., 1995). PEL express syndecan 1/CD138, which is a plasma cell surface marker, in addition to CD45 (Gaidano et al., 1997).

Although most PEL cell lines do not have translocations and mutations e.g., *c-myc* and *p53*, many PEL possess numerous genetic aberrations (Luan et al., 2010). Sophisticated comparative genome hybridizations (CGH) studies reveal extensive copy number aberrations comprising predominantly of gains and amplifications. Two genes, *SELPLG *and* CORO1C* were found to be the targets of amplification at chromosome 12q24.11. *SELPLG *encodes a membrane-bound glycoprotein that binds to P, E, and L-selectins, and is important for leukocyte recruitment to sites of inflammation (Laszik et al., 1996). *CORO1C* is a member of the coronin gene family that regulates actin-dependent processes such as motility and vesicle trafficking, and whose expression is associated with enhanced invasion and metastatic capability (Roadcap et al., 2008). Deletions of the two fragile site tumor suppressors, *WWOX* and *FHIT*, were also recently reported in PEL (Roy et al., 2011).

### MULTICENTRIC CASTLEMAN’S DISEASE

Multicentric Castleman’s disease, an atypical lymphoproliferative disorder, is divided into the hyaline vascular type, and the plasmablastic type. KSHV is associated with the plasma cell variant of MCD, which is multicentric in that several lymph nodes and the spleen are involved in disease. In the context of HIV infection, MCD is systemic, aggressive, and is associated with high fatality. KSHV genomes are detected in almost all HIV+ MCD cases, and ~50% of non-HIV+ cases of MCD (Soulier et al., 1995; Dupin et al., 2000). AIDS patients diagnosed with MCD suffer sustained fevers, weight loss, lymphadenopathy, and hepatosplenomegaly (Du et al., 2001). MCD frequently progresses to lymphoma or KS.

Multicentric Castleman’s disease is localized to the marginal zone of lymph nodes and the spleen. The germinal centers resemble follicular hyperplasia, and the mantle zone is generally intact and surrounded by mature KSHV-infected plasmablasts (Dupin et al., 1999; Katano et al., 2000; Parravicini et al., 2000). MCD cells resemble plasmablast or pre-plasma cells (Jenner et al., 2003). All KSHV-infected plasmablasts within the lesion exclusively express the λ light chain of IgM (Du et al., 2001, and the presence of λ light chains and absence of CD138 on MCD cells further suggests they originate from the infection of a less differentiated B cell (Hassman et al., 2011). Lymph nodes involved in MCD are characterized by germinal center expansion and vascular endothelial proliferation. MCD is characterized by elevated serum interleukin (IL)-6 levels in the patient (Yoshizaki et al., 1989). These elevated IL-6 levels, partially augmented by virally encoded IL-6 (vIL-6), create an inflammatory microenvironment which significantly contributes to the pathophysiology of MCD.

## KSHV ALTERS NORMAL B CELL PROLIFERATION AND DIFFERENTIATION, LEADING TO LYMPHOPROLIFERATIVE DISORDERS

Both MCD and PEL are associated with infection of a pre-terminally differentiated plasma cell. Gene expression arrays indicate that PEL have a plasma cell expression profile, and enhanced expression of genes involved in inflammation, adhesion, and invasion (Jenner et al., 2003, likely contributing to their malignant phenotype. MCD is characterized by the polyclonal expansion of KSHV-infected plasmablasts that exclusively express the λ light chain. No functional significance exists in whether a plasmablast bears either λ or κ, as the isotype exclusion is purely a function of order of light chain gene rearrangement.

It was unknown whether KSHV preferentially infected λ light-chain bearing B cells due to an inherent, yet unknown survival advantage to the virus/infected cell, or whether KSHV infection of a more undifferentiated cell (prior to light chain rearrangement) drove the expansion of λ-expressing B cells.

Hassman et al. (2011)attempted to address this question in a recent study in which they infected *ex vivo* suspensions of human tonsillar cells with purified KSHV. Despite the presence of various cell types, KSHV infection was shown to preferentially occur in B cells, as evidenced by LANA+ staining. The tonsillar cultures contained both κ- and λ-expressing B cells, however, all LANA+ staining was observed within the λ subset. Furthermore, infection with KSHV enhanced the proliferation of this IgMλ subset, which was further augmented by treatment with IL-6. KSHV-positive cells mirrored phenotypic characteristics of MCD cells, such as blasting morphology and increased expression of IgMλ, CD27, Ki67, and IL-6R.

This study also suggested that rather than naïve B cells, KSHV preferentially infects IgM memory B cells, resident within sub-epithelial regions of the tonsil and spleen. During plasmablast differentiation, IgM memory B cells acquire phenotypic markers such as Ki67+, IgM+, and CD27+, similar to MCD. This model is concordant with molecular and seroepidemiological data suggesting that the primary mode of KSHV transmission is via saliva (Vieira et al., 1997).

Plasmablast differentiation, including that of IgM memory B cells, can result from NF-κB activation, a signaling event that occurs during KSHV infection, and also during latency. The viral FADD-like IL-1β-converting enzyme (FLICE/caspase-8) inhibitory protein (vFLIP) activates NF-κB signaling in latently infected cells by associating with the IκB kinase (IKK) complex, and driving degradation of IκBα, and thus, NF-κB translocation into the nucleus. A consequence of NF-κB signaling is the promotion of cell survival and proliferation pathways. Ballon et al. (2011)constructed an inducible CD19- or Cγ1-driven conditional vFLIP knock-in mouse, which targeted vFLIP expression to various stages of B cell development. vFLIP expression in B cells was found to prevent germinal center development, Ig class switching, and affinity maturation; vFLIP expression resulted in splenomegaly in the mice. Although transgenic mice did not recapitulate phenotypes of PEL, B cells mimicked an MCD phenotype, with expansion of IgM λ plasmablasts, concordant with the findings of Hassman et al. (2011) indicating that vFLIP leads to expansion of the IgMλ subset upon KSHV infection. Moreover, impaired GC formation and class switch recombination resulting from vFLIP expression suggests that inhibition of the adaptive response is a means of escaping immune surveillance.

Interestingly, 20-month old vFLIP-expressing mice developed B cell-derived histiocytic/dendritic cell (DC) sarcoma. This finding is important as it underscores the ability of vFLIP to either reprogram or transdifferentiate the infected lymphocytes or the bystander cells in a paracrine manner, into other cell types. These findings also demonstrate the inherent plasticity of B lymphocytes (Cobaleda and Busslinger, 2008).

The collective body of research suggests that KSHV viral oncogenesis is mediated by expression of both latent and lytic viral gene products. Viral latent proteins are expressed in every tumor cell, whereas lytic proteins are expressed in a small percentage of tumor cells undergoing reactivation, and are thought to promote cell proliferation in an autocrine or paracrine manner. KSHV can infect a wide range of cell types *in vitro *and *in vivo *including monocytes, plasmacytoid DCs, fibroblasts, keratinocytes, B lymphocytes, endothelial, and epithelial cells (Kliche et al., 1998; Renne et al., 1998; Lagunoff et al., 2002; Akula et al., 2003; Inoue et al., 2003; Krishnan et al., 2004; Naranatt et al., 2004; Kaleeba and Berger, 2006a,b; Rappocciolo et al., 2006, 2008; Jarousse et al., 2008; Greene and Gao, 2009; Hassman et al., 2011; West et al., 2011; West and Damania, 2008). To guarantee successful replication within these distinct cell types, KSHV encodes an arsenal of viral proteins that are capable of modifying the host cell environment, either directly or indirectly, with the outcome being beneficial for the virus. Modulation of host cell pathways includes evasion of both immunity as well as apoptosis, induction of cell proliferation, and the promotion of cellular metabolism, macromolecular synthesis, and protein translation. One way KSHV accomplishes these alterations is by targeting the phosphatidylinositol-3-kinase (PI3K)/AKT/mammalian target of rapamycin (PI3K/AKT/mTOR) pathway. Similar to KSHV, many DNA viruses encode one or more viral proteins that either activate or inactivate various nodes of this pathway (Buchkovich et al., 2008).

## KSHV LIFE CYCLE

Kaposi sarcoma-associated herpesvirus establishes lifelong latency within the host, punctuated with sporadic bouts of reactivation and lytic replication. During latency, the KSHV genome exists as a circular, extra-chromosomal viral genome (episome), with minimal expression of a subset of latent viral proteins: K12/Kaposin, K13/vFLIP, ORF72/vCyclin, ORF73/LANA, vIL-6, and K1 (Wen and Damania, 2010b). In contrast, during the lytic replication phase, most of the viral genome is expressed and viral replication is followed by viral assembly, egress, and dissemination. The lytic switch protein replication and transcription activator (RTA) governs KSHV reactivation (Sun et al., 1998; Lukac et al., 1999). Chemicals such as phorbol esters, sodium butyrate, and histone deacetylase inhibitors reactivate the virus by activating the RTA promoter (Yu et al., 1999, and thus are useful for studying the viral life cycle. Hormones (norepinephrine and epinephrine), cytokines such as interferon-γ, oncostatin M, and hepatocyte growth factor reactivate KSHV, as does the hypoxic microenvironment typical of solid tumors and serous cavities, thus stimulating the expression of viral proteins that are beneficial to the host cell (Chang et al., 2000; Mercader et al., 2000). Terminal differentiation of B cells, resulting from expression of X-box binding protein 1 (XBP-1) can also activate the RTA promoter, inextricably linking virion production to the host cell life cycle (Wilson et al., 2007; Yu et al., 2007). Furthermore, stimulation of Toll-like receptors (TLRs) 7 and 8 by microbes can also reactivate KSHV from latently infected cells (Gregory et al., 2009; stimulation of these pattern recognition receptors generates an anti-viral state, and expressed lytic proteins have many functions which antagonize the host immune system.

Kaposi sarcoma-associated herpesvirus reactivation from latency significantly alters the physiology of the infected cell. Viral replication can reveal viral nucleic acid or peptide motifs that can activate host immune surveillance pathways. Viral replication also increases the demand for macromolecules such as nucleotides and amino acids to synthesize progeny virions. Cellular energy pools are substantially depleted in order to fuel the increased biosynthetic rates associated with viral replication. The combined effect of enhanced biosynthetic rates and unfulfilled energy demands is the activation of cellular stress responses, resulting in cell cycle arrest or apoptosis, in an attempt to resolve these deficits. To circumvent activation of stress responses, lytic proteins efficiently block cell death pathways, and maintain the host cell in a constant state of proliferation. Moreover, many lytic and latent proteins co-opt cellular signaling pathways, which sustain proliferation, block cell death, and enhance cellular metabolism, in order to maintain latent virus or facilitate lytic replication and dissemination of KSHV. One such pathway is the pleiotropic PI3K/AKT/mTOR pathway, which governs many cellular processes.

## THE PI3K/AKT/mTOR SIGNALING PATHWAY

The following brief introduction describes the various effectors of PI3K/AKT/mTOR signaling, and relates their activation to distinct physiological outcomes for the cell. While no means exhaustive, this description provides a primer for subsequent sections, which describe KSHV’s interaction with this pathway. For recent reviews describing up-to-date targets of PI3K/AKT/mTOR signaling, their regulation, and relevance to malignancies, please refer to Bunney and Katan (2010), Hsieh et al. (2011), Zoncu et al. (2011) and De Luca et al. (2012)

The PI3K are lipid kinases that phosphorylate the 3′-hydroxyl of the inositol ring of phosphoinositide, a component of the interior side of eukaryotic cell membranes (Engelman et al., 2006). Phosphoinositides help form membrane cell signaling complexes and are essential for intracellular trafficking. PI3K are divided into four classes, IA, IB, II, and III, all of which have differing substrate specificities and modes of regulation. Class IA and IB PI3K catalyze the phosphorylation of phosphatidylinositol-4,5-bisphosphate (PIP_2_) at the 3′ carbon on the inositol ring into phosphatidylinositol-3,4,5-triphosphate (PIP_3_). PI(3,4,5)P_3_ production by PI3K allows for pleckstrin homology (PH)-domain containing proteins to localize to the plasma membrane (Engelman et al., 2006). PIP_3_ also functions as a cellular second messenger capable of controlling cell shape, survival, proliferation, growth, and motility. Class I PI3Ks are heterodimeric proteins comprising a regulatory subunit and a catalytic subunit. The regulatory subunits are p85α, p85β, p55α, p55γ and p50α (Engelman et al., 2006). The catalytic subunits are comprised of one of four isoforms: p110α, p110β, p110δ and p110γ. Most mammalian tissues widely express p110α, β, and γ catalytic subunits, whereas p110δ is restricted to lymphocytes (Donahue and Fruman, 2004). Phosphatase and tensin homology (PTEN) is a phosphatase that catalyzes dephosphorylation of the 3′ carbon on the inositol of PI(3,4,5)P_3_ back to PIP_2_ (Cantley and Neel, 1999). PTEN is one of the most frequently lost tumor suppressors in various cancers.Mutations or deletions in *PTEN *cause hyperactivation of PI3K signaling, leading to increased cell proliferation as well as evasion of apoptosis. AKT is a PH-domain-containing protein that plays a central role in varied cellular processes such as glucose metabolism, evasion of apoptosis, and promotion of cell proliferation, transcription and cell migration (Manning and Cantley, 2007). AKT can also stimulate protein synthesis via activation of mTOR (discussed below; Hsieh et al., 2011). Once AKT is localized to the cell membrane through its PH-domain, it is phosphorylated on Threonine^308^ by phosphoinositide-dependent kinase 1 (PDK1), and serine^473^ through the mTORC2 complex (Lawlor and Alessi, 2001, both of which are activating modifications. AKT has several different effectors, which control distinct biological processes, thus, AKT activation has pleiotropic effects upon the cell. AKT inhibits cell death by phosphorylation-mediated inactivation of pro-apoptotic factors Bad, Caspase-9, and the FOXO group of transcription factors (Cross et al., 1995; Datta et al., 1997; del Peso et al., 1997; Cardone et al., 1998). Phosphorylation of the FOXO transcription factors sequesters them within the cytoplasm, thus preventing them from transcriptionally activating target pro-apoptotic genes such as Fas ligand and Bim.

AKT regulates the cell cycle by phosphorylating and inactivating key regulators of cell cycle progression. For instance, AKT promotes the transcriptional activation of *c-Myc* and *cyclin D1* genes (Gera et al., 2004, and interacts with two regulators of the cell cycle, p27 and p21 (Zhou et al., 2001; Shin et al., 2002). Maintaining a quiescent state requires high intracellular p27 levels, and PI3K activation was shown to reduce the cellular reserves of p27 (Sun et al., 1999). AKT phosphorylates the p53-induced protein p21, a negative regulator of cell cycle progression (Zhou et al., 2001). The net result of these inhibitory phosphorylation events is the maintenance of cell cycle progression and the de-regulation of cellular checkpoint signaling.

AKT also activates the non-canonical branch of the NF-κB family of transcription factors. NF-κB regulates many aspects of innate and adaptive immunity and cell survival. NF-κB is normally sequestered in the cytoplasm by inhibitory proteins such as inhibitor of κB (IκBα), which is degraded following phosphorylation by the upstream IKK complex, comprised of IKKα and IKKβ. IκBα is a direct target of IKKβ, that when degraded, releases NF-κB p65 thus activating genes involved in innate immune responses IKKα activates non-canonical NF-κB, leading to formation of p52 which activates genes involved in adaptive immunity (Verma et al., 1995; Ghosh et al., 1998; Karin and Ben-Neriah, 2000, and AKT regulates non-canonical NF-κB p52 processing by increasing the activity of IKKα (Gustin et al., 2006). Studies using constitutively active AKT demonstrated elevated p52 transcriptional activity. Non-canonical NF-κB activity was severely inhibited by using PI3K inhibitors, kinase-dead AKT or cells lacking AKT isoforms 1 or 2 (Gustin et al., 2006; Comb et al., 2012). Thus, AKT modulates both cell survival and regulation of adaptive immune responses. de Oliveira et al. (2010) provide an excellent review of the role of NF-κB signaling with regard to KSHV infection.

The serine/threonine kinase mTOR is a downstream target of PI3K/AKT signaling. In addition to being activated by essential signaling pathways such as PI3K and MAPK, mTOR is activated by a wide range of cellular stimuli such as growth factors, stress signals, and nutrient, energy, and amino acid abundance. mTOR activity is negatively regulated by the tuberous sclerosis complex (TSC), comprised of TSC1 and TSC2. TSC2 is an AKT target, and when phosphorylated, inhibits Rheb, also a negative regulator of mTOR (Manning and Cantley, 2007). mTOR exists in two distinct multiprotein signaling complexes, mTORC1 and mTORC2, which have differing sensitivities to the macrolide rapamycin; mTORC1 is sensitive, whereas mTORC2 is insensitive. mTORC1 regulates protein translation, cell size regulation, intracellular transport, metabolism, and lipid biogenesis. mTORC1 phosphorylates S6K1 and 4EBP1, which are two proteins critical for translation of eukaryotic capped mRNAs. S6K1 phosphorylates the S6 ribosome, thus stimulating protein synthesis. Unphosphorylated 4EBP1 tightly binds and represses the eukaryotic initiation factor 4E (eIF4E); hyperphosphorylated 4EBP1 releases eIF4E, thereby enabling cap-dependent translation (Gingras et al., 1999). mTOR has a wide plethora of other targets, for example ULK1 which regulates autophagy (Zoncu et al., 2011). Because protein translation is central to both cancer growth and viral persistence, mTOR is a very important signaling protein. The rapamycin-insensitive mTORC2 complex regulates cell survival and cytoskeleton dynamics. mTORC2 activates AKT, thereby paradoxically activating AKT/mTOR signaling even upon rapamycin treatment, demonstrating a feedback activation loop (Sarbassov et al., 2005).

Solid tumors are characterized by hypoxic microenvironments, therefore, *de novo* angiogenesis as well as remodeling existing blood vessels is essential to provide the rapidly growing cells with nutrients and oxygen. The viscosity and shear forces of blood against the walls of blood vessels govern the enzymatic activity of endothelial cell-expressed NOS (eNOS), and consequently, the continual synthesis and release of nitric oxide (NO). In endothelial cells, AKT is activated in a PI3K-dependent manner, both by shear forces and VEGF (vascular endothelial growth factor), which collectively activate eNOS (Dimmeler et al., 1999). NO has pleiotropic functions ranging from angiogenesis, remodeling of the vasculature, and the control of blood vessel tone (Thomas et al., 2008).

The AKT/mTOR axis is a critical regulator of cellular metabolism. Activated AKT stimulates glucose uptake by re-localizing the GLUT1 glucose transporter, thus bringing glucose into the cell for fueling various cellular processes (Wieman et al., 2007). Activation of the mTORC1 complex promotes glycolytic flux, up-regulates the pentose phosphate pathway, and stimulates *de novo* lipogenesis, all of which contribute to metabolic reprogramming essential for rapidly dividing cancer cells (Duvel et al., 2010).

As mentioned above, hyperactivation of PI3K/AKT/mTOR signaling is a characteristic of many malignancies (Kodaki et al., 1994; Samuels et al., 2004). Deregulated signaling may result from inactivation of negative regulator phosphatases, e.g., TSC2 or PTEN, or from mutations in catalytic domains of kinases, e.g., PIK3CA. Although KSHV-infected PEL cells are not known to have activating mutations in any of these three kinases, PI3K/AKT/mTOR signaling is highly up-regulated in these cells (Sin et al., 2007; Bhatt et al., 2010). As we will discuss further, a variety of viral proteins can activate this signaling pathway.

Proper PI3K/AKT/mTOR signaling is essential for the differentiation and developmental program of normal T and B lymphocytes, as well as other immune cells. PI3K is downstream of numerous cytokine receptors – CD40, TLRs, and the BCR itself. The current body of research suggests that PI3K signaling regulates the development of bone marrow B cell precursors, as well as the differentiation and development of B cell subsets (Hodson and Turner, 2009; Srinivasan et al., 2009; Beer-Hammer et al., 2010). Moreover, PI3K signaling also governs many aspects of activation and proliferation of mature B cells. For an excellent review describing the PI3K pathway with regard to B cell development, please see Donahue and Fruman (2004).

## KSHV ACTIVATES PI3K SIGNALING DURING *DE NOVO* INFECTION

Kaposi sarcoma-associated herpesvirus activates the PI3K signaling pathway during viral infection. The widely expressed proteoglycan heparan sulfate, α3β1 integrins, DC-SIGN, and xCT are the primary receptors for KSHV, and their differential distribution in various cell types contributes to the wide tropism of KSHV (reviewed in Chandran, 2010). KSHV enters target cells by endocytosis. Viral entry activates many cellular signaling pathways, which does not require active viral replication, as demonstrated by studies using UV-inactivated KSHV (Chandran, 2010). Viral ligation of cell-surface integrins triggers the phosphorylation and activation of focal adhesion kinase (FAK) in fibroblasts (Krishnan et al., 2006). FAK further activates downstream signaling molecules including Src, Rho GTPases, Diaphanous 2, and PI3K (Naranatt et al., 2003 ; Veettil et al., 2006; Raghu et al., 2007). In turn, these molecules further activate their own downstream effectors such as Ezrin, protein kinase C (PKC), MEK, NF-κB, ERK-1/2 and p38 MAPKs, and AKT.

Integrin-mediated tyrosine phosphorylation of FAK occurs minutes after KSHV infection of fibroblasts (Chandran, 2010). Reduced viral genomes and gene expression are observed in cells lacking either FAK or Pyk2, a FAK family member, illustrating their essential role in viral infection of target cells (Naranatt et al., 2003; Krishnan et al., 2006). FAK and Pyk2 signaling converge onto the Src kinase family, whose downstream effectors are PI3K and Rho GTPases. FAK, Src, and PI3K are also phosphorylated following infection of THP-1 monocytes, as are NF-κB and ERK-1/2 (Kerur et al., 2010). PI3K activation is crucial for *de novo* infection due to its activation of various GTPases involved in actin cytoskeleton remodeling, endosome formation, and vesicle trafficking. These intracellular processes allow for viral entry and delivery into the nuclear compartment. PI3K and Rho GTPase activation collectively induces other Rho GTPase family members, which precipitate the formation of subcellular structures such as lamellipodia (through Rac), stress fibers (through RhoA), and filopodia (through Cdc42; Krishnan et al., 2006). Further, activation of the PI3K target, AKT, leads to the inhibition of pro-apoptotic factors, thus protecting KSHV-infected cells from cell death.

Activation of cellular signaling is imperative for successful viral infection. The signaling nodes described above activate the processes of vesicle formation, intracellular motility, and evasion of cell death. Furthermore, transcription factors activated by these signaling pathways also play a role in activation of the viral transcription program, as well as induction of cellular proteins that facilitate viral replication. Thus, the collective activation of these intracellular signaling pathways creates an environment benefiting the KSHV life cycle.

## KSHV VIRAL PROTEINS THAT ACTIVATE PI3K/AKT/mTOR SIGNALING

Currently, four of the approximately 100 genes and microRNAs encoded by KSHV are known to impinge upon the PI3K/AKT/mTOR signaling pathway. They are K1, viral G protein-coupled receptor (vGPCR), vIL-6, and ORF45. We will discuss the mechanisms, extent, context and physiological relevance of each of these proteins below.

### K1

K1 is the first ORF encoded by KSHV, and is located at the extreme left end of the viral genome. K1 is a transmembrane glycoprotein whose expression in rodent fibroblasts induces morphological changes and the ability to grow in foci, indicating K1’s transformation capacity (Lee et al., 1998). Further, infection of T lymphocytes with a recombinant herpesvirus Saimiri expressing K1 instead of the oncoprotein, Saimiri transforming protein (STP), conferred IL-2 independent growth, suggesting that K1 is also an oncoprotein (Lee et al., 1998). All KSHV-associated tumors express low levels of K1 transcript, and K1 expression is highly up-regulated early during lytic replication (Lagunoff and Ganem, 1997; Jenner et al., 2001; Lee et al., 2003; Wang et al., 2006; Chandriani et al., 2010). K1 transgenic mice display constitutively active NF-κB and Oct-2 transcription factors, increase in expression of basic fibroblast growth factor (bFGF), as well as up-regulated expression and activity of the Lyn tyrosine kinase (Prakash et al., 2002). A physiological consequence of K1-mediated alteration of the cellular transcription program is the development of tumors similar to spindle-cell sarcomatoid tumor and malignant plasmablastic lymphoma (Prakash et al., 2002).

**FIGURE 1:**
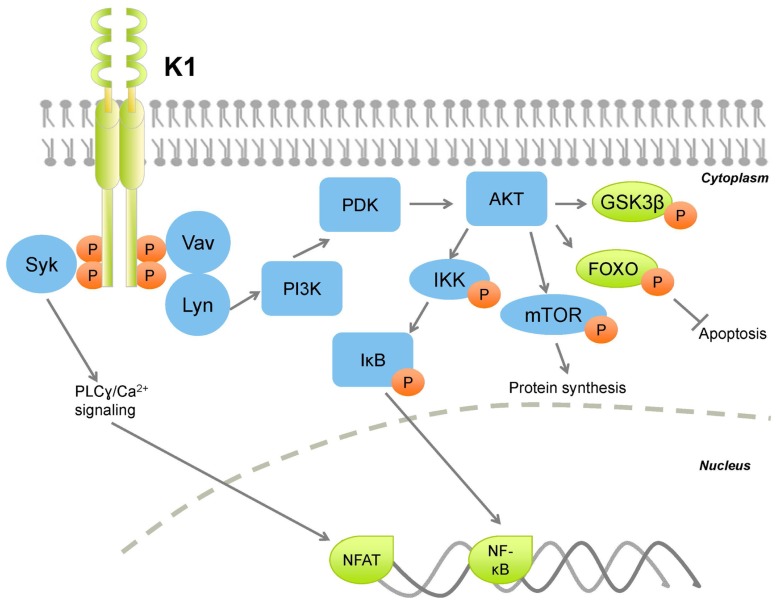
**Kaposi sarcoma-associated herpesvirus K1 activates PI3K/AKT/mTOR signaling thereby activating protein synthesis and survival pathways, while inhibiting apoptotic pathways**. Orange circles denote phosphorylation.

Various studies describe the extent to which K1 also deregulates normal cellular signaling (**Figure 1**). The regulatory p85 subunit modulates PI3K activity, and tyrosine-phosphorylation of p85 results in activation of PI3K (Cuevas et al., 2001). K1 expression leads to increased tyrosine phosphorylation of p85, in addition to phosphorylation of Vav and Syk, thus activating signaling networks downstream of these kinases, which have pleiotropic effects on the cell (Lagunoff et al., 1999; Lee et al., 2003; Tomlinson and Damania, 2004). Further, activation of transcription factors downstream of these kinases, for example, NFAT, downstream of Syk signaling, further augments deregulation of cellular signaling and promotes cell survival.

In B lymphocytes, ectopic K1 expression was found to activate AKT signaling in two simultaneous ways: K1 expression induced AKT phosphorylation on Thr^308^ and Ser^473^, and also inactivation of the negative regulator PTEN (Tomlinson and Damania, 2004). K1-mediated AKT activation induced the cytoplasmic sequestration of the FOXO family of transcription factors, and subsequent reduction of Fas ligand expression, thus conferring a cell survival advantage to K1-expressing cells (Tomlinson and Damania, 2004). K1 also stabilizes AKT through interaction with the cellular chaperones heat shock protein 90 β (Hsp90β) and the endoplasmic reticulum-associated Hsp40 (Erdj3/DnaJB11), (Wen and Damania, 2010a, both of which are important for enhancing the signaling function of AKT (Sato et al., 2000; Gao et al., 2003). Chaperone-mediated stabilization of AKT by K1 is essential for sustained signaling, as their inhibition induced caspase-3-dependent apoptosis in FasL-treated, K1-expressing cells (Wen and Damania, 2010a).

K1’s cytoplasmic tail contains an immunoreceptor tyrosine-based activation motif (ITAM; Lagunoff and Ganem, 1997; Lee et al., 2003). ITAMs are essential for signal transduction in immune cells, therefore are found on immunoreceptors, for example, CD79α and β, subunits of the B cell receptor complex. Upon ligand binding, the tyrosine residues on ITAMs are phosphorylated, which allow for docking of SH2 domain-containing molecules (**Figure 1**). Downstream transduction of the extracellular signal induces calcium mobilization from the endoplasmic reticulum, and activates the lymphocyte. K1 does not require ligand binding to induce signaling, and functions as a constitutively active receptor (Asmuth et al., 2003). The K1 ITAM is closely conserved across KSHV strains, indicating the importance of this motif for K1 function (Zong et al., 1999, 2002). The constitutive activity of the K1 ITAM activates a variety of downstream signaling pathways that not only protect the infected cell, but also neighboring cells in a paracrine fashion. Notably, K1 also activates PI3K/AKT/mTOR signaling in endothelial cells (Wang et al., 2004, 2006). Components of the K1 signalosome have been identified, and indicate that the K1 ITAM interacts with a diverse set of cellular signaling proteins (Lee et al., 2005). Overall, K1 interactions with cellular proteins augments global cellular signal transduction, activation of transcription facts such as NF-κB and AP-1, and induction of inflammatory cytokines (Lee et al., 2005).

Interactions of the K1 N-terminal domain with the BCR complex induces BCR sequestration within the endoplasmic reticulum (Lee et al., 2000). Because normal BCR signaling can potentially induce apoptosis, BCR sequestration preempts this possibility, thus conferring a long-term survival advantage to the infected cell.

K1 expression is up-regulated during viral reactivation from latency. Lytic replication may induce pro-apoptotic signals resulting from immune detection of replicating KSHV. Viral replication also places increased demands for energy and nutrients on the cell (Munger et al., 2006, and induces a stress response that can activate apoptosis. These collective pro-apoptotic signals can be subverted by K1 expression (Tomlinson and Damania, 2004; Wen and Damania, 2010a, thereby supporting productive lytic replication and further dissemination of KSHV. Moreover, PI3K activation can also re-start protein translation and metabolic programs, halted as a result of apoptotic signals, ensuring that raw materials for production of progeny virions are plentiful.

### KSHV vGPCR

Kaposi sarcoma-associated herpesvirus ORF74 encodes a vGPCR, a seven-pass transmembrane protein homologous to the cellular IL-8 chemokine receptor (Cesarman et al., 1996). vGPCR is a ligand-independent receptor expressed during the lytic cycle. Genes expressed during lytic replication are potently transforming, as they exert strong survival signals to prolong the life of the host cell, which ultimately dies as a result of viral replication and associated cellular stress. vGPCR has transforming properties; it promotes focus formation in mouse NIH3T3 cells, and vGPCR-expressing cells form tumors in nude mice (Bais et al., 1998). Human umbilical vein endothelial cells (HUVECs) are immortalized by vGPCR expression, and also protected from apoptosis induced by serum-starvation (Montaner et al., 2001). In various mouse models, vGPCR expression leads to formation of vascular tumors and KS-like angioproliferative lesions, with cell surface markers and circulating cytokine profiles resembling KS (Yang et al., 2000; Guo et al., 2003; Montaner et al., 2003).

Being a constitutively active receptor protein, vGPCR can function without the need for ligand binding (Rosenkilde et al., 1999). However, vGPCR is capable of binding members of both CXC and CC chemokine families (Sodhi et al., 2004). Some CXC chemokines, such as GRO-α and IL-8, enhance vGPCR signaling (Rosenkilde et al., 2000, whereas interferon γ-inducible 10-kDa protein (IP-10/CXCL10) and stromal cell-derived factor 1 α (SDF-1α) inhibit vGPCR signaling (Gershengorn et al., 1998). IL-8 is a major mediator of inflammation, and recruits neutrophils, basophils, and T cells. IL-8 released during the immune response to lytic KSHV replication may enhance the function of vGPCR in lytically infected cells, thereby inducing an anti-apoptotic signal to delay the death of the infected cell. More puzzling is why SDF-1/CXCL12, a stimulator of B cell progenitor proliferation, inhibits vGPCR activity (Gershengorn et al., 1998). However, since vGPCR is primarily expressed during the lytic cycle, this might not have a consequence for latently infected B cells.

**FIGURE 2:**
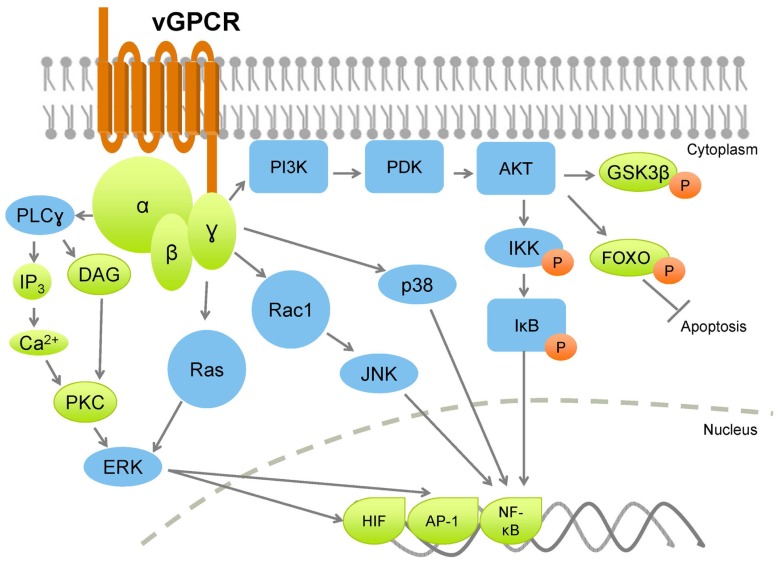
**Kaposi sarcoma-associated herpesvirus vGPCR broadly activates PI3K and MAPK pathways, leading to increased production of cytokines and growth factors, with a concurrent increase in cell proliferation, and inhibition of apoptotic pathways**. Orange circles denote phosphorylation.

**FIGURE 3:**
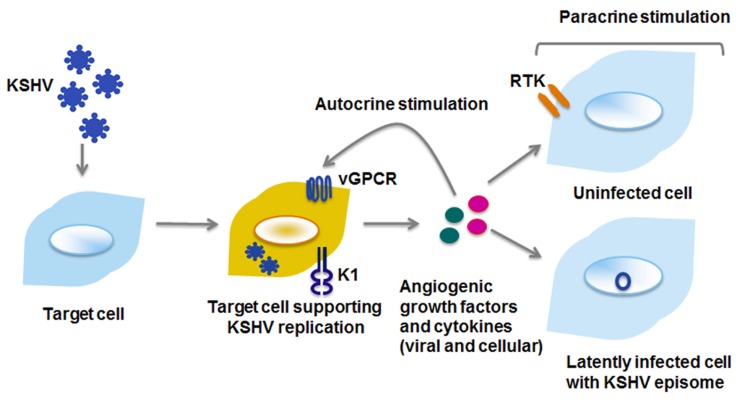
**Viral proteins enhance cell proliferation by autocrine and paracrine mechanisms**. Viral and cellular cytokines and growth factors can activate signaling pathways within the cell they are secreted from (autocrine), or on distant cells that may either be uninfected or latently infected with KSHV (paracrine). RTK, receptor tyrosine kinase.

KSHV vGPCR activates a plethora of cellular signaling molecules as well as transcription factors, by means of which it promotes transformation in endothelial, epithelial, and fibroblast cells (**Figure 2**). vGPCR activates pathways such as PLC/PKC, Pyk2/Lyn, ERK, p38, and JNK, downstream of which are transcription factors that control many growth- and angiogenesis-promoting genes. For example, HIF-1α activation resulting from vGPCR-dependent p38 and MAPK signaling activates the VEGF promoter (Sodhi et al., 2000). vGPCR also activates AP-1, NF-AT, and NF-κB transcription factors, which in turn promote expression of a panoply of pro-inflammatory cytokines, growth factors, and adhesion molecules (Couty et al., 2001; Montaner et al., 2001; Schwarz and Murphy, 2001; Pati et al., 2003). The vGPCR-mediated secretion of such a wide array of factors may enhance proliferation and survival in neighboring, uninfected cells in a paracrine manner (**Figure 3**). Indeed, a recent report demonstrates that cytokines secreted by a small number of vGPCR-positive tumor cells activate signaling pathways in neighboring cells, converging on mTOR-dependent VEGF up-regulation (Jham et al., 2011). Inhibiting paracrine mTOR activity in non-expressing cells abrogated the tumor-promoting activities of vGPCR-expressing cells *in vivo*. On the other hand, vGPCR expression may directly induce transformation of the cell, due to up-regulated signaling resulting from the same secreted factors. These two methods of transformation may act in concert, rather than in isolation, leading to transformation of both the infected and bystander cells.

PI3K/AKT/mTOR signaling is a common pathway downstream of many growth factor and cytokine receptors. In particular, the tissue-restricted γ isoform of PI3K is crucial for relaying vGPCR signaling downstream to AKT/mTOR in endothelial cells (Martin et al., 2011). Paracrine secretions resulting from vGPCR expression activate PI3K/AKT/mTOR signaling (e.g., VEGF), and moreover, vGPCR directly activates AKT in a PI3K-dependent manner (Montaner et al., 2001).

vGPCR expression in the B cell neoplasms, PEL and MCD, exhibits a distinct gene expression profile compared to endothelial cells (Polson et al., 2002, and is also characterized by elevated PI3K/AKT and ERK/p38 MAPK signaling. Ectopic vGPCR expression in B cells activates several transcription factors: AP-1, CREB, NF-AT, and NF-κB, thereby promoting cell survival, although the mechanisms of activation of these transcription factors differ (Cannon et al., 2003).

As mentioned above, although capable of constitutive activity, vGPCR can also signal by coupling with cellular Gα_q_ and Gα_i_ subunits (Cannon and Cesarman, 2004, further amplifying PI3K/AKT signaling, as both Gα_q_ and Gα_i_ subunits signal through this pathway (Murga et al., 1998). Additionally, vGPCR-mediated activation of AP-1 and CREB (but not NF-κB and NFAT) in B cells was found to be dependent on PI3K/AKT (Cannon and Cesarman, 2004). vGPCR also activates endogenous Lyn tyrosine kinase in a Gα_i_- and PI3K-dependent manner. Pharmacologic and genetic ablation of Src family kinases abolished AP-1 and CREB transcriptional activity, confirming that these transcription factors are activated by vGPCR through a Gα_i_-PI3K/AKT-Src signaling axis. Further, this study showed that Src inhibitors decreased AKT phosphorylation in PEL, indicating that in B cells, Src may be upstream rather than downstream of PI3K/AKT signaling, suggestive of a positive feedback loop. Importantly, this study revealed that NF-κB and NF-AT are not activated in a PI3K/AKT-dependent manner in B cells (Cannon and Cesarman, 2004). Subsequent studies indicated that the Ras-related small G protein Rac1 may be required for NF-κB activation *via* vGPCR (Montaner et al., 2004).

The previous paragraphs describe the extent to which vGPCR activates cellular signaling. The lytically replicating, vGPCR-expressing cell activates transcription factors and signaling entities within the infected cell; induction of secreted factors further amplifies signaling in neighboring cells, with the collective outcome of enhanced proliferation and sustained survival (**Figure 3**).

### VIRAL IL-6

Viral IL-6, encoded by ORF-K2, is a homolog of the human IL-6 (hIL-6) cytokine, with 24.8% amino acid sequence similarity and 49.7% sequence identity (Moore et al., 1996). Functionally, vIL-6 is a faithful mimic of hIL-6, as vIL-6 secreted by KSHV-infected B lymphocytes supports proliferation of B lymphocytes and also hIL-6-dependent mouse myeloma cell lines, demonstrating its functional similarity (Moore et al., 1996; Neipel et al., 1997; Nicholas et al., 1997). Most latently infected cells express low levels of vIL-6, with up-regulated expression during lytic replication (Cannon et al., 1999; Staskus et al., 1999; Parravicini et al., 2000; Chandriani et al., 2010). Similar to its cellular counterpart, vIL-6 signaling activates the JAK/STAT, MAPK, and H7-sensitive pathways (Osborne et al., 1999). Although vIL-6 and hIL-6 have similar sequence and function, their receptor usage is substantially different. Cellular IL-6 requires two IL-6 receptor subunits: IL-6Rα and gp130. However, vIL-6 is capable of signaling via only the gp130 subunit (Molden et al., 1997). Thus, KSHV vIL-6 circumvents the requirement for a second receptor, thereby subverting cellular checks against uncontrolled, exuberant signaling.

vIL-6 is implicated as a linchpin in the pathology of all KSHV-associated malignancies, due to its angiogenic properties, as well as potent proliferative and survival effects. vIL-6 is detected, in increasing order, in KS, PEL, and MCD patients (Aoki et al., 2000, 2001). However, only a subset of cells express vIL-6 in KS tissue (Cannon et al., 1999; Staskus et al., 1999; Parravicini et al., 2000, suggesting that like vGPCR, vIL-6 effects are primarily paracrine, activating proliferative signaling pathways in bystander cells. Similarly, in MCD, vIL-6 is expressed in lymphoid cells in mantle zones, and hIL-6 was detected in germinal centers (Cannon et al., 1999; Staskus et al., 1999; Parravicini et al., 2000).

Injection of vIL-6-expressing NIH3T3 cells into athymic mice led to tumor formation, hematopoiesis, and plasmacytosis, all of which were absent in control mice. Tumors derived from vIL-6-expressing cells were highly vascularized, and correlated with elevated secreted VEGF (Aoki and Tosato, 1999). A recent report of transgenic mice constitutively expressing vIL-6 describes elevated serum vIL-6, increased levels of phospho-STAT3 levels in the spleen and lymph nodes, and a manifestation of human MCD-like symptoms (Suthaus et al., 2012). Importantly, when vIL-6 was constitutively expressed in a mouse lacking endogenous hIL-6, no MCD-like symptoms were observed. These studies indicate that vIL-6 and hIL-6 cooperate in the pathogenesis of MCD.

Lymphatic reprogramming resulting from KSHV infection of endothelial cells also occurs via engagement of the gp130 receptor (Morris et al., 2008). Specifically, gp130 receptor activation leads to the activation of JAK2/STAT3 and PI3K/AKT pathways. Activated AKT promotes the expression of Prox1, a lymphatic transcription factor necessary for VEGFR-3 induction, and Prox1 itself is known to potentiate the lymphatic reprogramming of endothelial cells upon *de novo *KSHV infection (Hong et al., 2004). Furthermore, STAT3 amplifies this signaling cascade by activating gp130 receptor expression. Podoplanin, another marker of lymphatic reprogramming, is also expressed following KSHV activation of gp130. While not found to be necessary, vIL-6 is sufficient to induce lymphatic reprogramming in endothelial cells infected with KSHV (Morris et al., 2012). Thus, through gp130, vIL-6 can enhance lymphangiogenesis and lymphatic reprogramming in both a paracrine and autocrine manner.

### ORF45

ORF45 is an immediate early gene product that plays a crucial role in lytic replication. ORF45 expression is induced upon entry into the lytic cycle and subsequently increases as the life cycle advances, with expression restricted to the cytoplasm. ORF45 is incorporated into the KSHV virion (Zhu and Yuan, 2003, suggesting that it may immediately influence the environment of the *de novo* infected host cell, exemplified by the observation that ORF45 acutely inhibits type 1 IFN induction upon infection. ORF45 mediates the inhibition of innate immune responses by sequestering the cellular transcription factor, interferon regulatory factor-7 (IRF-7), to the cytoplasm (Zhu et al., 2002).

In addition to the modulation of cellular anti-viral responses, ORF45 also exerts an effect upon the cellular signaling *milieu*. Cellular MAPKs are activated during KSHV infection (Sharma-Walia et al., 2005; Xie et al., 2005, and ORF45 interacts with two important MAPK substrates RSK1 and RSK2, which are both p90 ribosomal S6 kinase (RSK) family members. RSK1 and 2 not only phosphorylate ORF45, but their association further augments the kinase activities of these two proteins (Kuang et al., 2008, 2009).

Another consequence of formation of the RSK/ERK/ORF45 complexes is phosphorylation of ribosomal protein S6, and eIF4B, an important member of the complex that recruits ribosomes to 5′ capped mRNAs. Phosphorylated eIF4B complexes with eIF4A, eIF4G, 4E-BP1, and 5′ capped mRNAs to recruit the 40S ribosome, thereby initiating protein translation. Normally, S6 and eIF4B are activated by p70 S6 kinase, itself regulated by upstream mTOR signaling; eIF4B is also a target of the p90 S6 kinase, regulated by MAPK signaling. However, ORF45 allows for eIF4B phosphorylation in an mTOR- and MAPK-independent manner. These observations indicate that protein translation may occur upon KSHV infection in an mTOR-independent manner. They also demonstrate the existence of unique viral strategies to directly enhance protein translation despite a situation within the host cell that may potentially be inhibitory to protein translation.

## KSHV-MEDIATED TRANSFORMATION AND THE HALLMARKS OF CANCER

The hallmarks of cancer are a conceptual framework to understand the multistep progression of cancer (Hanahan and Weinberg, 2000, 2011). The hallmarks of cancer take into account that neoplasms, rather than being a singular, isolated entity, are a collection of distinct cell types, comprised of tumor cells, tumor-associated stroma as well as normal, non-cancerous cells. These diverse cell types cooperate to collectively confer hallmark capabilities, which further enhance the development of a tumor microenvironment. Acquisition of one hallmark by a normal cell facilitates the development of others, thus increasing the likelihood of cellular transformation. The classical hallmarks of cancer are sustained proliferation signaling, evasion of growth suppression, replicative immortality, induction of angiogenesis, evasion of cell death, and invasion and metastasis. Three additional hallmarks have also been recently proposed: deregulation of cellular energetics, tumor-promoting inflammation, and avoidance of immune recognition.

The endpoint of the productive KSHV life cycle is the production of new virions that can subsequently infect new cells, and begin another round of the viral life cycle. To successfully replicate and generate progeny virions, KSHV must evade immune recognition during latency, and also counter antiviral and death-inducing signaling during lytic replication. A plethora of viral proteins successfully grant invisibility from immune recognition, and prolong the life of the infected cell by altering cellular signaling, as described above.

### SUSTAINED PROLIFERATIVE SIGNALING

Proliferation is a carefully controlled process in normal cells. Uncontrolled growth would lead to nutrient scarcity as well as overgrowth in the physical niche. KSHV encodes genes that activate cellular proliferative pathways both in a paracrine and autocrine fashion (**Figure 3**; Lagunoff et al., 1999; Lee et al., 2003; Tomlinson and Damania, 2004). This hallmark is particularly evident in KS, as not all cells within the lesion are KSHV-positive; expression of viral and cellular factors bestows neighboring cells with enhanced proliferative capabilities. Sustained proliferation results from growth factors binding to their cognate cell surface receptors, and subsequent signaling that further promotes expression of the same growth factors and receptors, in a positive feedback loop.

### EVASION OF GROWTH SUPPRESSION AND CELL DEATH

Powerful growth inhibitory programs are intrinsic to the host cell to prevent uncontrolled proliferation. Hyperproliferation can trigger cell senescence via activation of checkpoint signaling. Alternatively, apoptosis may also result from aberrant oncogene activation. Immune cells that detect virally infected cells may also transduce apoptotic signals to facilitate viral clearance.

KSHV encodes several proteins to protect the infected cell from these growth suppressive and cell death-inducing signals, during latency and the lytic cycle. For example, vCyclin, a homolog of cellular cyclin D, forces quiescent rodent cells to enter the S-phase to overcome RB-mediated growth arrest induced by CDK inhibitors (Swanton et al., 1997). In the same vein, cells supporting lytic replication do not die prematurely despite increased cellular stress, due to the presence of viral proteins that inhibit apoptotic signaling. As described above, K1-dependent, AKT-mediated sequestration of FOXO transcription factors and inactivation of Bad can protect B cells from apoptosis (Tomlinson and Damania, 2004). Similarly, vFLIP expression also protects cells from apoptosis by up-regulating NF-κB transcription and pro-survival factors (Sun et al., 2003; Matta and Chaudhary, 2004; Thurau et al., 2009). In the context of the whole virus, KSHV-infected primary HUVEC cells are more resistant to apoptotic stimuli such as etoposide, staurosporine, and serum-starvation, compared to uninfected cells (Wang and Damania, 2008). Thus, evasion of both cell death and growth suppression by viral proteins expressed during latency and the lytic cycle contribute to the development of KSHV-associated cancers.

### REPLICATIVE IMMORTALITY

Somatic cells within the body divide a finite number of times, i.e., they have limited replicative potential. Upon reaching their limit, normal cells senesce and cannot proliferate any further. The number of cell divisions is governed in part by telomeres, which are stretches of repetitive DNA at the ends of chromosomes that shorten after every cell division. However, cancer cells can replicate indefinitely owing to activation of one of two pathways: activation of human telomerase (hTERT), or the activation of an alternative (ALT) pathway, both of which lead to lengthening of telomeres. KSHV LANA has been shown to increase the expression of the catalytic subunit of telomerase, hTERT by up-regulating its promoter, thereby contributing to replicative immortality (Knight et al., 2001). Additionally, K1 expression in primary HUVECs endows replicative immortality, primarily through the ALT pathway (Wang et al., 2006).

### INDUCTION OF ANGIOGENESIS

All cells, whether normal or cancerous, require a reliable blood supply to provide nutrients and oxygen, and to eliminate carbon dioxide and metabolic waste products. Tumor cells promote formation of vasculature by activating angiogenesis, as well as remodeling of existing vasculature and sprouting new vessel growth. Often, tumor-associated vessels are erratically branched, aberrantly sized, excessively convoluted and structurally unsound, all resulting from hyperactivated induction of angiogenic factors (Baluk et al., 2005; Nagy et al., 2010). VEGF is a key mediator of angiogenesis, and its expression is governed by upstream signaling pathways such as PI3K/AKT (Jiang and Liu, 2009). Other inflammatory cytokines can also drive angiogenesis. KSHV vGPCR induces VEGF and VEGF receptor 2 secretion in endothelial cells (Bais et al., 2003). Moreover, K1 expression in epithelial and endothelial cells also induces secretion of VEGF and the invasion factor matrix metalloprotease-9 (MMP-9; discussed below), as does KSHV infection of endothelial cells (Wang et al., 2004.

### INVASION AND METASTASIS

Invasion and metastasis is a multistep process that begins with local invasion due to cancer cells exceeding their occupied niche, resulting from hyperproliferation. Cancer cells that weakly adhere to neighboring cells easily escape and intravasate nearby blood vessels and lymph nodes and travel to distal sites through vasculature and lymphatics (Langley and Fidler, 2011). Some cells escape these vessels and enter a new environment, often substantially different than the first, forming micrometastases; in the final stage, these grow into larger masses that colonize the new niche, generating metastases (Langley and Fidler, 2011). Activation of a developmental regulatory program termed “epithelial–mesenchymal transition” (EMT) bestows onto epithelial cells the ability to invade and metastasize (Thiery, 2002; Mani et al., 2008). EMT includes a transcriptional and signaling program, and a similar, “endothelial to mesenchymal” (EndMT) transition occurs in the context of KSHV infection (Cheng et al., 2011). vFLIP and vGPCR activate the Notch signaling pathway, resulting in secretion of the mesenchymal marker membrane type-1 MMP (MT1-MMP), granting invasive properties to KSHV-positive cells. Significantly, MT1-MMP was found co-localized with LANA in KS biopsies. These data suggest that the presence of heterogeneous cell types within KS lesions can result from viral proteins driving EndMT within infected cells, bestowing them with invasion capabilities, and the creation of a microenvironment that benefits viral dissemination.

### DEREGULATION OF CELLULAR ENERGETICS

Recent evidence suggests that the reprogramming of cellular energetics and metabolism is an emerging hallmark of cancer (reviewed in Hanahan and Weinberg, 2011). Fueling uncontrolled proliferation and cell division of tumor cells requires rewiring of normal cellular energetics. Normal cells, in aerobic conditions, utilize glucose to first generate pyruvate and ATP by glycolysis, and subsequent mitochondrial oxidative phosphorylation (OXPHOS). Anaerobic conditions result in a switch to glycolysis, which is relatively inefficient and generates smaller quantities of ATP, which may or may not be accompanied by reduced OXPHOS. Warburg observed that cancer cells preferentially oxidize glucose by glycolysis even in aerobic conditions, limiting their energy production; this phenomenon is termed the Warburg effect, or aerobic glycolysis (Warburg et al., 1924). The Warburg effect is an adaptation of tumors growing in hypoxic conditions to generate ATP. KSHV infection of endothelial cells induces the Warburg effect, and glycolysis inhibition of latently infected cells leads to apoptosis (Delgado et al., 2010). Moreover, we reported that in KSHV-infected PEL, aerobic glycolysis fuels *de novo* lipid synthesis to generate precursors for daughter cells, explaining the significance of up-regulating an energetically unfavorable biochemical process (Bhatt et al., 2012). This study also demonstrated that glycolysis and fatty acid synthesis (FAS) occur in a PI3K/AKT-dependent manner, providing a mechanism for metabolic reprogramming in PEL. Further, PEL viability was found to be susceptible to FAS inhibitors, revealing a new molecular therapeutic target.

### IMMUNE EVASION

An ever-watchful immune system surveys the body for signs of nascent neoplasms, and eliminates such cells. The ability to escape immune surveillance is a frequent consequence of genetic instability and aberrant signaling in tumors. KSHV-associated tumors are even more adept at hiding from the immune system as viral protein expression can subvert various aspects of the innate and adaptive immune response. Viral proteins, e.g., KSHV vIRFs, K3, K5, etc. inhibit immune signaling, protecting the infected cell from host detection. For example, the K3 and K5 viral proteins can down-regulate both class I and II major histocompatibility complexes (MHC), enhancing the immunoevasion capabilities of infected cells (Coscoy and Ganem, 2000; Ishido et al., 2000). The KSHV vIRFs also contribute to immune evasion (reviewed in Jacobs and Damania, 2011). As discussed in previous sections, apoptotic signaling resulting from immune detection is also potently inhibited by viral protein expression.

### TUMOR-PROMOTING INFLAMMATORY MICROENVIRONMENT

Similar to non-viral tumors, KSHV-associated lesions are infiltrated by a large number of immune cells. KSHV-associated neoplasms are also characterized by elevated local and systemic levels of inflammatory cytokines and chemokines, further augmented by virally encoded cytokines such as vIL-6, vMIPs/vCCls, and vOX2. KSHV infection up-regulates cyclooxygenase-2 (COX-2), an enzyme that converts arachidonic acid into prostaglandins, which are inflammation mediators (Sharma-Walia et al., 2010). COX-2 is essential for survival of KSHV-infected cells, and viral genome maintenance, both of which are susceptible to COX-2 pharmacological inhibitors. Creation of an inflammatory environment is functionally significant, since it activates signaling in surrounding tissues, and recruits readily infectable cell types to facilitate viral dissemination.

## EXPLOITING THE PI3K/AKT/mTOR PATHWAY TO TREAT KSHV-ASSOCIATED MALIGNANCIES

Individual KSHV proteins can activate PI3K/AKT/mTOR signaling in B cells and endothelial cells, and this pathway is important for both lytic and latent phases of the KSHV life cycle. Additionally, both KS and PEL display highly activated AKT and mTOR kinases (Montaner et al., 2001; Uddin et al., 2005; Sin et al., 2007). Because aberrant PI3K/AKT/mTOR signaling is a characteristic of almost all human cancers, a plethora of small molecule inhibitors exist that target various nodes of this pathway. These inhibitors include allosteric inhibitors such as rapamycin and FK506, and also ATP-competitive small molecule kinase inhibitors that usually target the kinase activity of specific proteins.

Rapamycin is a macrolide that binds to FKBP12, a component of the mTOR signaling complex (mTORC), thus making it an allosteric inhibitor (Sawyers, 2003). Rapamycin is commonly used as an oral immunosuppressant for solid organ transplant recipients, as it inhibits the production and secretion of IL-2 in T cells, thus blocking T cell proliferation. Moreover, rapamycin blocks protein translation. Therefore, rapamycin and its derivative compounds called “rapalogs” are extensively studied for their therapeutic benefit in a variety of human cancers, including those associated with viral infection (Dittmer et al., 2012). Rapamycin treatment resolved transplant-associated KS (Stallone et al., 2005, a seminal finding that has prompted many other studies which confirm that rapamycin is an effective anti-cancer drug for PEL (Sin et al., 2007). Specifically, rapamycin is effective at halting the proliferation of PEL in cell culture, and in a xenograft model of PEL, rapamycin inhibits tumor formation and induces tumor regression (Sin et al., 2007). One drawback of rapamycin therapy is that it slows tumor growth (tumorstatic), rather than killing tumor cells (tumortoxic). Therefore, single agent therapy with rapamycin alone has limited benefit in a majority of cancers.

A class of AKT inhibitors called alkyl-lysophospholipids (e.g., miltefosine and perifosine) also inhibited PEL cell proliferation both *in vitro* and *in vivo *(Bhatt et al., 2010). Moreover, NVP-BEZ235, a dual inhibitor of both PI3K and mTOR kinases, is a potent inhibitor of PEL cell proliferation and tumor formation in xenograft mouse models. NVP-BEZ235 treatment induced high levels of apoptosis in PEL (Bhatt et al., 2010). Thus, it appears that the PI3K/AKT/mTOR signaling pathway is essential for the survival of both PEL and KS tumors. It is of critical importance to evaluate whether long-term treatment with small molecule inhibitors breeds resistance to pathway-focused inhibitors. Selective pressure resulting from these inhibitors could drive expression of viral proteins that may contribute to resistance. Therefore in the future, it will be important to investigate whether as yet uncharacterized KSHV proteins influence PI3K/AKT/mTOR signaling, both in the context of latency and lytic viral replication.

## CONCLUSION

Kaposi sarcoma-associated herpesvirus is an obligate intracellular parasite, and is a salient example of a successful pathogen. Viral manipulation of cellular pathways enhances the synthesis and secretion of growth factors and cytokines of both viral and cellular origin, which in turn support angiogenesis and proliferation. These secreted growth factors and cytokines can also activate pro-survival, proliferative, and angiogenic processes in uninfected or latently infected cells (**Figure 3**). Thus, by manipulating cellular signaling, KSHV viral proteins create an environment beneficial for both lytic and latent phases of the viral life cycle. Given the central role it plays in cell survival and proliferation, it comes as no surprise that KSHV targets the PI3K/AKT/mTOR signaling pathway at multiple nodes, in order to induce and sustain a survival and proliferative signal that is advantageous for the virus.

## Conflict of Interest Statement

The authors declare that the research was conducted in the absence of any commercial or financial relationships that could be construed as a potential conflict of interest.
